# Supplementary material for: ProteinShader: illustrative rendering of macromolecules
Source: BMC Struct Biol. 2009 Mar 30;9:19. doi: 10.1186/1472-6807-9-19 (PMC2672931; doi:10.1186/1472-6807-9-19)
Supplement: Additional file 1 — ProteinShader program without source code. This compressed file contains the complete ProteinShader program including associated libraries, but no source code. A README.txt file gives an overview of the ProteinShader distribution, and the index.html file in the help subdirectory has directions on getting started with the program as well as a set of tutorials. [file 1472-6807-9-19-S1.zip › ProteinShader-beta-0_9_4-binary/help/api/org/proteinshader/structure/class-use/Chain.html]

Uses of Class org.proteinshader.structure.Chain (ProteinShader API)


|  |  |  |
| --- | --- | --- |
| **Overview** \| **Package** \| **Class** \| **Use** \| **Tree** \| **Deprecated** \| **Index** \| **Help** | |  |
| PREV   NEXT | **FRAMES**    **NO FRAMES**     **All Classes** |  |


---


## **Uses of Class org.proteinshader.structure.Chain**

| Packages that use Chain | |
| --- | --- |
| **org.proteinshader.gui** | Holds all of the Swing GUI components and their associated listeners, including class Renderer, which is registered as a listener for the GLCanvas object that is used a drawing surface. |
| **org.proteinshader.gui.components.controlpanel** | Holds the JPanels used in the right-side retractable control panel of the GUI. |
| **org.proteinshader.structure** | Holds the classes that store information from a Protein Data Bank file: Structure, Model, Chain, AminoAcid, Heterogen, Water, Atom, Bond, Helix, BetaStrand, Loop, *etc*. |
| **org.proteinshader.structure.visitor** | Holds Visitor classes that know how to traverse the hierarchy of objects held by class Structure. |

Uses of Chain in org.proteinshader.gui

| Methods in org.proteinshader.gui that return Chain | |
| --- | --- |
| `Chain` | `MediatorImpl.getCurrentChain()`             Returns the current Chain. |
| `Chain` | `Mediator.getCurrentChain()`             Returns the current Chain. |

| Methods in org.proteinshader.gui with parameters of type Chain | |
| --- | --- |
| `void` | `MediatorImpl.setCurrentChain(Chain chain)`             Sets the current Chain. |
| `void` | `Mediator.setCurrentChain(Chain chain)`             Sets the current Chain. |

Uses of Chain in org.proteinshader.gui.components.controlpanel

| Methods in org.proteinshader.gui.components.controlpanel that return Chain | |
| --- | --- |
| `Chain` | `SelectorPanel.getSelectedChain()`             Returns the currently selected Chain from the Chain menu. |

| Methods in org.proteinshader.gui.components.controlpanel with parameters of type Chain | |
| --- | --- |
| `void` | `SelectorPanel.setResidueList(Chain chain)`             Checks the currently selected Residue type (AminoAcids, Heterogens, or Waters) and copies them from the Chain to the Residue list. |

Uses of Chain in org.proteinshader.structure

| Methods in org.proteinshader.structure that return Chain | |
| --- | --- |
| `Chain` | `Model.addNewChain(String chainID)`             Creates a new Chain with the chainID given as an argument, adds the Chain to the Model's collection of Chains, and returns a reference to the new Chain. |
| `Chain` | `Model.getChain(String chainID)`             Returns the Chain with the chainID given as an argument. |
| `Chain` | `Structure.getChain(String chainID, String modelID)`             Returns the Chain with the IDs given as arguments. |

| Methods in org.proteinshader.structure that return types with arguments of type Chain | |
| --- | --- |
| `Iterator<Chain>` | `Model.iteratorChains()`             Returns an Iterator for the Chains held by this Model. |

Constructors in org.proteinshader.structure with parameters of type Chain

`BetaStrand(String betaStrandID,
String betaStrandNumber,
String sheetID,
String startResidueID,
String endResidueID,
Chain chain,
int sense,
int strandsInSheet)`
  
          Constructs a BetaStrand.

`Helix(String helixID,
String serialNo,
String startResidueID,
String endResidueID,
Chain chain,
HelixEnum type,
HelixShapeEnum shape)`
  
          Constructs a Helix.

`Loop(String loopID,
String startResidueID,
String endResidueID,
Chain chain)`
  
          Constructs a Loop.

`Region(String startResidueID,
String endResidueID,
Chain chain)`
  
          Constructor for use by subclasses.

Uses of Chain in org.proteinshader.structure.visitor

| Methods in org.proteinshader.structure.visitor with parameters of type Chain | |
| --- | --- |
| `void` | `Visitor.visit(Chain chain)`             Uses the current ResidueMode to determine if it should traverse the AminoAcids, Heterogens, and/or Waters owned by the Chain. |
| `void` | `SFWriterVisitor.visit(Chain chain)`             Writes all information held by a Chain. |
| `void` | `FrenetFrameGeneratorVisitor.visit(Chain chain)`             All AminoAcids of the Chain will be visited. |
| `void` | `LoopGeneratorVisitor.visit(Chain chain)`             Generates Loop objects from AminoAcids that do not already belong to any Region. |
| `void` | `BondGeneratorVisitor.visit(Chain chain)`             Generates all standard Bonds (AminoAcids and Waters) for the Chain. |

---


|  |  |  |
| --- | --- | --- |
| **Overview** \| **Package** \| **Class** \| **Use** \| **Tree** \| **Deprecated** \| **Index** \| **Help** | |  |
| PREV   NEXT | **FRAMES**    **NO FRAMES**     **All Classes** |  |


---

# *Copyright © 2007-2008*
